# Team Korea injury and illness surveillance at the 2024 Paris Olympic Games

**DOI:** 10.1186/s40621-025-00610-z

**Published:** 2025-09-02

**Authors:** Junghyun Bae, Sukil Kim, Seungrim Yi, Jungjin Yu, Hyunchul Kim, Seungsoo Woo, Hyoungjoo Choi, Seyong Kim

**Affiliations:** 1 Medical Center, Jincheon National Training Center of the Korean Sport and Olympic Committee, 105, Seonsuchon-ro, Gwanghyewon-myeon, Chungcheongbuk-do, Jincheon-gun, 27809 Republic of Korea; 2 Department of Preventive Medicine, College of Medicine, The Catholic University of Korea, 222, Banpo-daero, Seocho-gu, Seoul, 06591 Republic of Korea; 3 Department of Pyeongchang National Training Support, Pyeongchang National Training Center of the Korean Sport and Olympic Committee, 108-27, Olympic-ro, Daegwallyeong-myeon, Gangwon-do, Pyeongchang-gun, Republic of Korea

**Keywords:** Epidemiology, Athlete, Athletic injuries, Disease, Incidence

## Abstract

**Background:**

The purpose of this study was to describe the injury and illness epidemiology in Team Korea athletes during 2024 Paris Summer Olympic Games. Incidence rates in pre-Olympic training camp and the Olympic Village were calculated and compared.

**Methods:**

Medical records of Team Korea athletes who competed for 2024 Paris Summer Olympic Games were analyzed. This study was conducted in accordance with the International Olympic Committee Consensus Statement 2020. Incidence rates (case per 1000 athlete-days) were calculated and incidence rate ratios were used for comparisons.

**Results:**

Team Korea athletes (*n* = 122, 57 males and 65 females) were analyzed. A total of 43 injuries (pre-Olympic training camp, *n* = 6, 8.3 injuries per 1000 athlete-days; Olympic Village, *n* = 37, 22.9 injuries per 1000 athlete-days) and 53 illnesses (pre-Olympic training camp, *n* = 16, 22.1 illnesses per 1000 athlete-days; Olympic Village, *n* = 37, 22.9 illnesses per 1000 athlete-days) newly occurred. The Olympic Village had a greater risk of injury than the pre-Olympic training camp (incidence rate ratio = 2.8, 95% CI: 1.2 to 6.6, *p*-value = 0.016). However, there was no significant difference in the risk of illness (incidence rate ratio = 1.04, 95% CI: 0.58 to 1.87, *p*-value = 0.900).

**Conclusions:**

Team Korea athletes participating in the 2024 Paris Summer Olympic Games exhibited higher incidences of injury and illness than previous Olympic Games statistics. This single-delegation epidemiological research will contribute to our understanding of the true incidence of health problems in Olympians.

**Supplementary Information:**

The online version contains supplementary material available at 10.1186/s40621-025-00610-z.

## Background

Most frameworks or models applied to methods for preventing injuries and illnesses to protect the health of athletes emphasize injury and illness surveillance [1, 2]. Such surveillances have been conducted at the Olympic Games over the past two decades [3–8]. The International Olympic Committee (IOC) has been trying to ensure consistency in the definition, data collection, and methodology in the surveillance by publishing consensus statements [9, 10]. These surveillances have played an important role in identifying patterns and hypotheses of athletes’ health problems [11, 12].

Like IOC [11] and other National Olympic Committees (NOCs) [13, 14], the Korean Olympic Committee (KOC) [15] also prioritizes protection of the health of athletes and seeks to establish a systematic injury and illness surveillance system. The KOC operated the pre-Olympic training camp (OTC) at the London Olympic Games 2012. However, no official report on injury or illness surveillance was published. The surveillance report from the OTC at Paris 2024 would help us prepare for the next OTC at the LA 2028 Summer Olympic Games.

This study aimed to describe epidemiologic characteristics of injuries and illnesses from Team Korea athletes in the KOC clinics of the pre-Olympic training camp (OTC) and in the Olympic Village (OLV) based on novel IOC consensus statement [10] during the 2024 Paris Summer Olympic Games.

## Methods

### Study design

Medical records documented during Paris 2024 Olympic Games and officially approved by the KOC were retrospectively analyzed in this observational study. We conducted this study in accordance with the IOC consensus statement and the Strengthening the Reporting of Observational Studies in Epidemiology for Sport Injury and Illness Surveillance (STROBE-SIIS) Statement 1.0 [10]. This study was approved by the Institutional Review Board (IRB) of The Catholic University of Korea, Catholic Medical Center (IRB approval number: MC24RISI0122/2024-2479-0001).

### Equity, diversity and inclusion statement

Our study included Team Korea athletes competed in Paris 2024 (all Asian, males = 45.8%, females = 54.2%) and no specific efforts were made to recruit participants. They were provided with medical support regardless of education or socioeconomic backgrounds. Our research team included eight men, medical support personnel who participated in Paris 2024 (three sports physician and four physiotherapist) and one epidemiologist.

### Setting

The KOC operated the OTC at the National Center of Defense Sports (Centre National des Sports de La Défense, Camp Guynemer, Fontainebleau) from July 12 to August 10, 2024 and the OLV during the Games from July 20 to August 12, 2024.

### Data acquisition

Three experienced KOC team physicians (each with over 15 years of experience and IOC recording training) documented medical encounters. The first author checked data daily, and final diagnoses and severity were confirmed by consensus. Data was recorded in a shared Google Sheet adhered to IOC 2020 guidelines with drop-down menus for consistency.

### Participants

All Team Korea athletes who competed in Paris 2024 and obtained support from the medical team at the OTC and/or OLV were participants of this study [see Additional file 1].

### Variables

Injuries and illnesses were classified with the Orchard Sports Injury and Illness Classification System (OSIICS revised 2020 consensus versions) [16]. New injuries and illnesses during the study period were included in this study. Injuries and illnesses in the same body region or organ system within the preceding three months were excluded. Injuries that occurred during competition, training, and peri-competition activities were included. However, injuries unrelated to sports activities such as falls in accommodation were excluded. Multiple injuries in a single event were individually documented but counted as a unique incidence.

Time-loss duration was adopted to present severity of an injury. If an injured or ill athlete was able to fully compete or train the next day, then time-loss duration was recorded as 0. Severity was stratified into 0 day (no time-loss), 1–7 days (mild), 8–28 days (moderate), and > 28 days (severe). Burden was defined as the time-loss duration divided by exposure. The longest time-loss duration was selected for calculating burden of multiple injuries or illnesses. When the time-loss duration was expected to be longer than the remaining competition period, the estimation was recorded according to clinical judgement based on findings of ultrasounds or Magnetic Resonance Imaging (MRI) scans.

### Data reduction and statistical methods

Records were pseudonymized by the KOC administrator so that individual athletes could not be identified. An epidemiologist participated in the overall research process.

Descriptive statistics are expressed as mean ± standard deviation for continuous variables with normal distribution or as frequencies and percentages for nominal variables. Incidence was calculated with number of injuries or illnesses as a numerator and respective number of exposed athletes and time exposure (athlete-days) as a denominator. Records of athletes held by the KOC were used to calculate accurate exposure. Incidence rates in the OLV were compared with those in the OTC.

Excel (Microsoft Corporation, Redmond, Washington, USA) and R (Ver. 4.4.1, R Core Team, Vienna, Austria) were used for all statistical analyses. Confidence intervals for incidence were calculated using the W*ilson* and *Byar* method of the *epi.conf* function with the package *EpiR*. Ratio was analyzed using the *rateratio* function of the package *fmsb*.

## Results

Team Korea Olympic delegation comprised 144 athletes (66 males and 78 females; mean age, 25.9 ± 4.8 years) across 21 sports in Paris 2024.

### Athletes with injury and/or illness

This study included 97 athletes (43 males and 54 females) who entered the OTC from July 12 to August 10, 2024 across 13 sports. Subsequently, 122 athletes (57 males and 65 females) in 18 sports were provided with medical support at the OLV from July 20 to August 12, 2024. Athletes spent an average of 6.6 ± 6.0 days in the OTC and 11.4 ± 4.6 days in the OLV.

A total of 240 health problems were reviewed. Of them, 144 health problems were excluded from the analysis, including 141 pre-existing health problems and 3 records with an unclear past medical history. Finally, 43 (6 in OTC and 37 in OLV) injuries and 53 (16 in OTC and 37 in OLV) illnesses were identified. A total of 30 (24.6%, 30/122) athletes in both OTC and OLV sustained at least one injury, while 37 (30.3%, 37/122) athletes experienced at least one illness. Eight athletes in the OLV had multiple injuries and nine athletes in the OLV had multiple illnesses.

### Incidence of injury and illness

The OLV had a higher risk of injury than the OTC (incidence rate ratio = 2.8, 95% CI: 1.2 to 6.6, *p*-value = 0.016). There was no difference in the risk of illness between OTC and OLV (incidence rate ratio = 1.0, 95% CI: 0.6 to 1.9, *p*-value = 0.900) (Table 1). There were no significant differences in injury or illness incidence rate between male and female athletes in OTC (injury incidence rate ratio = 0.4, 95% CI: 0.1 to 2.4, *p* = 0.325; illness incidence rate ratio = 1.9, 95% CI: 0.7 to 5.5, *p* = 0.218) or OLV (injury incidence rate ratio = 1.4, 95% CI: 0.7 to 2.7, *p* = 0.319; illness incidence rate ratio: 1.0, 95% CI: 0.5 to 1.9, *p* = 0.994).

**Table 1:** Incidence of injuries and illnesses in team Korea athletes competing at Paris 2024

	Sex	Total number of injuries or illnesses	Total number of athletes	Number of athletes with an injury or illness	Total number of athlete-days	Athletes with an injury or illness (%)	Injuries or illnesses per 100 athletes (95% CI)	Injuries or illnesses per 1000 athlete-days (95% CI)
Injuries of the OTC	ALL	6	97	6	725	6.2	6.2 (2.9 to 12.8)	8.3 (3.4 to 17.1)
	Male	4	43	4	338	9.3	9.3 (3.7 to 21.6)	11.8 (4.0 to 28.1)
	Female	2	54	2	387	3.7	3.7 (1.0 to 12.5)	5.2 (1.0 to 16.6)
Injuries of the OLV	ALL	37	122	27	1615	22.1	30.3 (22.9 to 39.0)	22.9 (16.4 to 31.2)
	Male	14	43	10	743	23.3	32.6 (20.5 to 47.5)	18.8 (10.8 to 30.8)
	Female	23	54	17	872	31.5	42.6 (30.3 to 55.8)	26.4 (17.2 to 38.9)
Illnesses of the OTC	ALL	16	97	14	725	14.4	16.5 (10.4 to 25.1)	22.1 (13.1 to 35.0)
	Male	5	57	5	338	8.8	8.8 (3.8 to 18.9)	14.8 (5.6 to 32.4)
	Female	11	65	9	387	13.8	16.9 (9.7 to 27.8)	28.4 (15.1 to 49.1)
Illnesses of the OLV	ALL	37	122	27	1615	22.1	30.3 (22.9 to 39.0)	22.9 (16.4 to 31.2)
	Male	17	57	14	743	24.6	29.8 (19.5 to 42.7)	22.9 (13.8 to 35.8)
	Female	20	65	13	872	20	30.8 (20.9 to 42.8)	22.9 (14.5 to 34.7)

OTC = the pre-Olympic training camp, OLV = the Olympic Village

### Injury by mode of onset, mechanism, and type

**Fig. 1:**
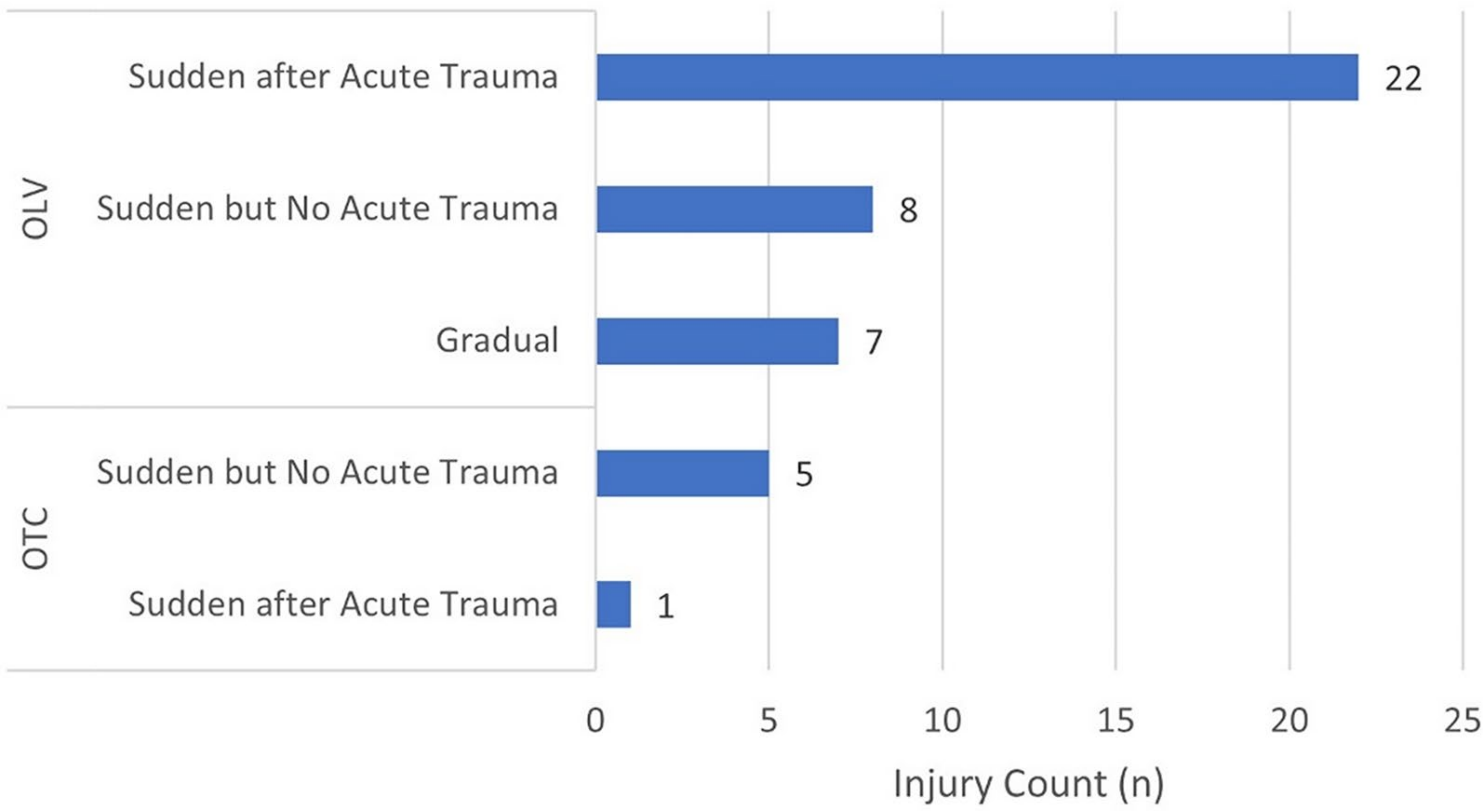
Number of injuries by mode of onset in Team Korea athletes competing at Paris 2024. Abbreviations: OTC = the pre-Olympic training camp, OLV = the Olympic Village

**Fig. 2:**
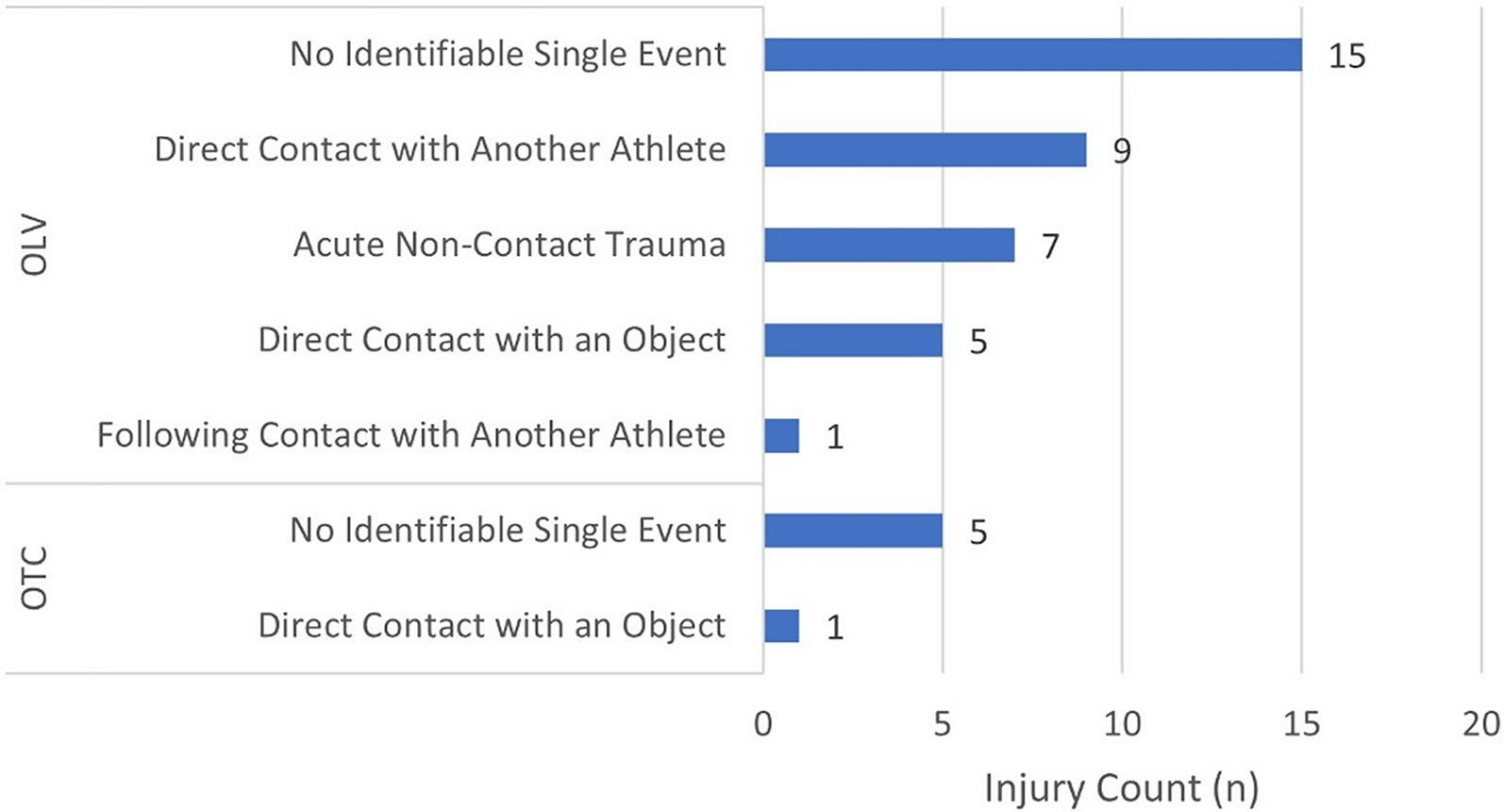
Number of injuries by injury mechanism in Team Korea athletes competing at Paris 2024. Abbreviations: OTC = the pre-Olympic training camp, OLV = the Olympic Village

**Fig. 3:**
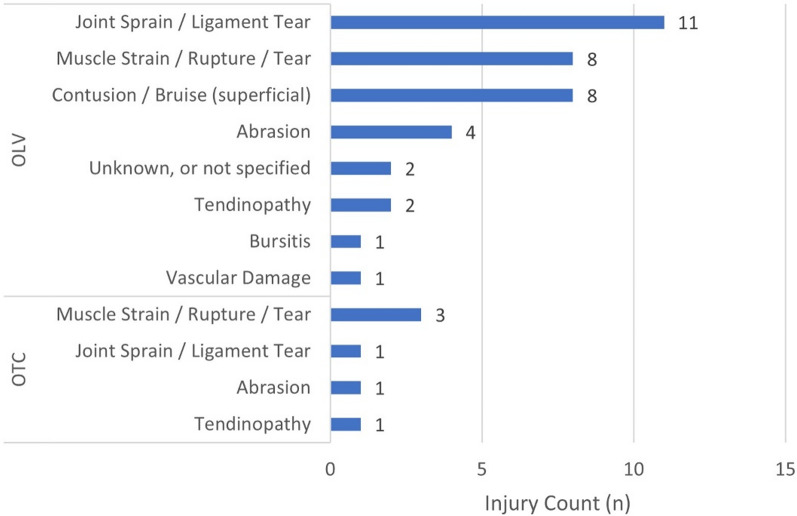
Number of injuries by injury type in Team Korea athletes competing at Paris 2024

All injuries in the OTC occurred during training. About two-thirds of injuries in the OLV occurred during competition (62.2%, 23/37). The remaining one-third occurred during training (32.4%, 12/37) and peri-competition periods (5.4%, 2/37). Most (83.3%, 5/6) injuries occurred suddenly without acute trauma in the OTC, whereas the greatest number of injuries (59.5%, 22/37) resulted from sudden acute trauma in the OLV (Fig. 1). One-third of acute traumas were caused by direct contact with another athlete (31.8%, 7/22) (Fig. 2). In the OTC, muscle strain/rupture/tear was the most prevalent injury type (50.0%, 3/6). In the OLV, joint sprain/ligament tear was the most common (29.7%, 11/37) (Fig. 3).

###  Injury incidence by body region

Regarding injury incidence by body region, lower limb injuries (54.1%, 20/37) in the OLV were more frequent than torso (18.9%, 7/37) and upper limb (24.3%, 9/37) injuries (Table 2). Four thigh injuries were reported among three athletes, with one athlete sustaining two separate injuries.

**Table 2:** Injury counts and incidence by body region in team Korea athletes competing at Paris 2024

Facility/Body region		Total number of injuries	Injuries per 100 athletes (95% CI)	Injuries per 1000 athlete-days (95% CI)
OTC (*n* = 97)	All	6	6.2 (2.9 to 12.8)	8.3 (3.4 to 17.1)
	Head/face/neck/cervical spine	0	-	-
	Head/face	0	-	-
	Neck/cervical spine	0	-	-
	Torso	1	1.0 (0.2 to 5.6)	1.4 (0.1 to 6.4)
	Chest	0	-	-
	Thoracic spine/upper back	0	-	-
	Lumbar-sacral spine/buttock	1	1.0 (0.2 to 5.6)	1.4 (0.1 to 6.4)
	Abdomen	0	-	-
	Hip/groin	0	-	-
	Upper limb	1	1.0 (0.2 to 5.6)	1.4 (0.1 to 6.4)
	Shoulder	0	-	-
	Upper arm	0	-	-
	Elbow	0	-	-
	Forearm	0	-	-
	Wrist	1	1.0 (0.2 to 5.6)	1.4 (0.1 to 6.4)
	Hand	0	-	-
	Lower limb	4	4.1 (1.6 to 10.1)	5.5 (1.8 to 13.1)
	Thigh	1	1.0 (0.2 to 5.6)	1.4 (0.1 to 6.4)
	Knee	0	-	-
	Lower leg/Achilles tendon	1	1.0 (0.2 to 5.6)	1.4 (0.1 to 6.4)
	Ankle	1	1.0 (0.2 to 5.6)	1.4 (0.1 to 6.4)
	Foot	1	1.0 (0.2 to 5.6)	1.4 (0.1 to 6.4)
OLV (*n* = 122)	All	37	30.3 (22.9 to 39.0)	22.9 (16.4 to 31.2)
	Head/face/neck/cervical spine	1	0.8 (0.1 to 4.5)	0.6 (0.1 to 2.9)
	Head/face	1	0.8 (0.1 to 4.5)	0.6 (0.1 to 2.9)
	Neck/cervical spine	0	-	-
	Torso	7	5.7 (2.8 to 11.4)	4.3 (1.9 to 8.5)
	Chest	2	1.6 (0.5 to 5.8)	1.2 (0.2 to 4.0)
	Thoracic spine/upper back	1	0.8 (0.1 to 4.5)	0.6 (0.1 to 2.9)
	Lumbar-sacral spine/buttock	2	1.6 (0.5 to 5.8)	1.2 (0.2 to 4.0)
	Abdomen	1	0.8 (0.1 to 4.5)	0.6 (0.1 to 2.9)
	Hip/groin	1	0.8 (0.1 to 4.5)	0.6 (0.1 to 2.9)
	Upper limb	9	7.4 (3.9 to 13.4)	5.6 (2.7 to 10.2)
	Shoulder	5	4.1 (1.8 to 9.2)	3.1 (1.2 to 6.8)
	Upper arm	0	-	-
	Elbow	1	0.8 (0.1 to 4.5)	0.6 (0.1 to 2.9)
	Forearm	1	0.8 (0.1 to 4.5)	0.6 (0.1 to 2.9)
	Wrist	1	0.8 (0.1 to 4.5)	0.6 (0.1 to 2.9)
	Hand	1	0.8 (0.1 to 4.5)	0.6 (0.1 to 2.9)
	Lower limb	20	16.4 (10.9 to 24.0)	12.4 (7.8 to 18.8)
	Thigh	**†**4	3.3 (1.3 to 8.1)	2.5 (0.8 to 5.9)
	Knee	3	2.5 (0.8 to 7.0)	1.9 (0.5 to 5.0)
	Lower leg/Achilles tendon	4	3.3 (1.3 to 8.1)	2.5 (0.8 to 5.9)
	Ankle	3	2.5 (0.8 to 7.0)	1.9 (0.5 to 5.0)
	Foot	6	4.9 (2.3 to 10.3)	3.7 (1.5 to 7.7)

OTC = the pre-Olympic training camp, OLV = the Olympic Village

**†** Three athletes sustained four thigh injuries

### Illness incidence by organ system and etiology

The OLV had a greater risk of respiratory illnesses than the OTC (incidence rate ratio = 3.3, 95% CI: 1.0 to 11.0, *p*-value = 0.04). The gastrointestinal system (31.3%, 5/16) was the most frequent site of illness in the OTC, followed by the respiratory system (18.8%, 3/16) and the dermatologic system (18.8%, 3/16). In the OLV, the respiratory system (59.5%, 22/37) was the most frequent site of illness, followed by the dermatologic system (21.6%, 8/37) and gastrointestinal system (2.7%, 1/37) (Table 3).

**Table 3:** Illness counts and incidence by organ system in team Korea athletes competing at Paris 2024

	Organ system	Total number of illnesses	Illnesses per 100 athletes (95% CI)	Illnesses per 1000 athlete-days (95% CI)
OTC (*n* = 97)	All	16	16.5 (10.4 to 25.1)	22.1 (13.1 to 35.0)
	Dermatological	3	3.1 (1.1 to 8.7)	4.1 (1.1 to 11.0)
	Dental	1	1.0 (0.2 to 5.6)	1.4 (0.1 to 6.4)
	Gastrointestinal	5	5.2 (2.2 to 11.5)	6.9 (2.6 to 15.1)
	Neurological	1	1.0 (0.2 to 5.6)	1.4 (0.1 to 6.4)
	Ophthalmological	1	1.0 (0.2 to 5.6)	1.4 (0.1 to 6.4)
	Psychiatric/psychological	1	1.0 (0.2 to 5.6)	1.4 (0.1 to 6.4)
	Respiratory system	3	3.1 (1.1 to 8.7)	4.1 (1.1 to 11.0)
	Unknown, or not specified	1	1.0 (0.2 to 5.6)	1.4 (0.1 to 6.4)
OLV (*n* = 122)	All	37	30.3 (22.9 to 39.0)	22.9 (16.4 to 31.2)
	Dermatological	8	6.6 (3.4 to 12.4)	5.0 (2.3 to 9.3)
	Dental	2	1.6 (0.5 to 5.8)	1.2 (0.2 to 4.0)
	Gastrointestinal	1	0.8 (0.1 to 4.5)	0.6 (0.1 to 2.9)
	Genitourinary	2	1.6 (0.5 to 5.8)	1.2 (0.2 to 4.0)
	Neurological	1	0.8 (0.1 to 4.5)	0.6 (0.1 to 2.9)
	Respiratory system	22	18.0 (12.2 to 25.8)	13.6 (8.8 to 20.3)
	Unknown, or not specified	1	0.8 (0.1 to 4.5)	0.6 (0.1 to 2.9)

OTC = the pre-Olympic training camp, OLV = the Olympic Village

Infection (47.2%, 25/53) was the main etiology across the OTC and the OLV. Notably, no gastrointestinal infection was observed. In the OLV, acute respiratory infection was the most frequent health problem (21 cases in 22 respiratory illnesses, 95.5%). No cardiovascular, endocrine, hematologic, musculoskeletal, otological, or thermoregulatory events were reported.

### Injury and illness by sports

Regarding injury and illness by sports in the OLV, judo (84.2 injuries/1000 athlete-days, 95% CI: 39.7 to 159.0) and artistic gymnastics (83.3 injuries/1000 athlete-days, 95% CI: 41.2 to 152.1) had the highest incidence rates of injuries while table tennis (43.5 illnesses/1000 athlete-days, 95% CI: 18.1 to 90.0) and judo (42.1 illnesses/1000 athlete-days, 95% CI: 14.1 to 100.1) had the highest incidence rates of illnesses.

### Severity

There were six time-loss injuries, 2 in the OTC (durations of 1 and 7 days) and 4 in the OLV (including a severe injury that occurred only once). Two injuries recorded durations of 1 and 2 days in the OLV. Another two injuries occurred in a single athlete at the end of the competition with estimated time-loss of 42 and 14 days based on ultrasound and MRI findings.

### Burden

The injury burden was 10.2 days per athlete-year in the OLV and 4.0 days per athlete-year in the OTC. There was no time-loss due to illness.

## Discussion

Epidemiologic characteristics in Team Korea athletes competed for the Paris 2024 were revealed in this surveillance. We found a total of 43 injuries and 53 illnesses. Injury and illness incidence of the Team Korea athletes during 2024 Paris Summer Olympic Games was more than twice those of previous Olympic Games [3–8]. This result aligned with results of other single-delegation studies [17–20]. The OLV had a 2.8 times greater risk of injury than the OTC. However, there was no significant difference in illness between OLV and OTC.

### Injury

In the OLV, 22.1% (*n* = 27) of athletes sustained at least one injury (30.3 injuries per 100 athletes, 22.9 injuries per 1000 athlete-days), which was approximately double the injury incidence reported in previous Olympic Games studies of 8–12% (9.1–12.9 injuries per 100 athletes, 5.4–7.8 injuries per 1000 athlete-days) [3–8]. A study that investigated injury and illness in Team Great Britain at Sochi 2014 also reported a relatively high incidence of 39% (48.2 injuries per 100 athletes) [19]. However, the Sochi 2014 Organizing Committee reported an incidence of 12% (14.0 injuries per 100 athletes, 7.8 injuries per 1000 athlete-days) [4]. Similarly, a subsequent study on injury and illness surveillance for Team USA at the Beijing 2022 revealed an incidence of 20.8% (30.3 injuries per 100 athletes; 16.5 injuries per 1000 athlete days) [17]. The Rio 2016 Team Korea epidemiologic study reported a substantial incidence proportion of 56.3% (151 injuries per 100 athletes) [20].

In the Tokyo 2020 study, its methodology suggested possible overestimation of exposure while potentially underestimating incidence [7]. Since it is difficult to verify all athletes in each NOC at the Olympic Games, cumulative incidence was used. This method assumed that the total number of athletes was constant during the Games when calculating exposure. Although previous studies on paralympic games have suggested that the variance in daily team size during the competition period is not large [21, 22], the Olympic Games period is longer than the 12-day Paralympic Games. Thus, some variations might be observed. Since schedules of Team Korea athletes were systematically managed by KOC, accurate exposure could be obtained. In our study, if we assumed that 122 athletes stayed constantly throughout 17 days, then exposure was calculated to be 2074 athlete days. However, the actual collected exposure was 1615 athlete days, which was about 78% of the calculated one. There might also be some underreporting from NOCs with smaller size and poor medical resources, although the number of all athletes was not large (53% of NOCs at Beijing 2022; 94 countries did not submit daily reports) [8].

The proportion of time-loss injury should also be noted in this study. In the case of previous Olympic Games, it was 33–44% [5–8]. Among the 37 OLV injuries, time-loss was 10.8% (*n* = 4). The previously mentioned Team Great Britain at Sochi 2014 also reported a low proportion of time-loss injuries of 7% [19]. For Team Korea at Rio 2016 [20], time-loss injuries accounted for 12%. At the 2018 Jakarta and Palembang Asian Games, it was 11.3% [18]. In this single delegation study, we could infer that less “serious” injuries requiring medical attention were collected in more detail, although there was no time-loss. Thus, this study might more closely reflect the real incidence.

In Rio 2016 [20] where the OTC was not operated, 64.0% of injuries were reported to have occurred during training, whereas 62.2% of OLV injuries occurred during competition in our study. We assumed that the presence of the OTC affected the incidence by reducing training sessions in the OLV.

Reports from the Rio 2016 and Tokyo 2020 committees indicate that boxing and BMX commonly see high injury incidences [5, 7]. However, Team Korea had no boxing injuries in Paris 2024 and did not participate in BMX at Rio, Tokyo, or Paris. Only a few Korean athletes competed in boxing: two in Paris and one in Rio.

Previous studies found higher injury incidences in taekwondo and handball than in judo and artistic gymnastics [5, 7]. However, at Paris 2024, Team Korea had no taekwondo injuries, and handball’s rate was less than half that of judo and gymnastics. Due to small sample sizes, these differences may be due to chance. Thus, we only report incidence rates by sport.

### Illness

As with injuries in the OLV, 22% (*n* = 27) of athletes sustained at least one illness (30.3 illnesses per 100 athletes; 22.9 illnesses per 1000 athlete-days), which was higher than the 4–9% (3.8–9.4 illnesses per 100 athletes, 2.3–5.5 illnesses per 1000 athlete-days) reported in previous Olympic Games [3–8], but similar to previous studies on illness among Team Korea athletes, including those reported for Rio 2016 (24.0%; 35.8 illnesses per 100 athletes) and the 2018 Jakarta-Palembang Asian Games (21.7%; 26.7 illnesses per 100 athletes) [18, 20].

Acute respiratory infections in athletes account for most of acute respiratory illnesses [23]. Paris 2024 was the first Summer Olympic Games after the COVID-19 pandemic. There was no mitigation policy for respiratory infection. Respiratory illnesses emerged as the predominant health concern in the OLV, accounting for 59.5% of total illnesses in our surveillance, similar to those in Rio 2016 (47%) [5]. The proportion of infection-induced respiratory illnesses remained consistently high: 95.5% in our surveillance and 76% in Rio 2016. In contrast, Tokyo 2020 reported that respiratory illnesses accounted for 17% of total illnesses, with 60% of cases caused by infection [7]. An interesting observation from these findings was that Team Korea athletes in the OTC exhibited decreased susceptibility to respiratory infections. Team Korea had no official campaigns or strategies to mitigate respiratory problems. The OTC is a French military facility that is closed to the public. Additionally, all athletes had their own accommodations.

A study on the Rio 2016 Team Korea also reported that respiratory illnesses accounted for 75.9% of total illnesses [20]. A study found a ten-fold drop in acute respiratory infections during the pandemic, crediting COVID-19 measures like masks, better hand hygiene, physical distancing, and smaller room occupancy [24]. Thus, Team Korea should introduce evidence-based prevention programs for respiratory illnesses including acute respiratory infections.

### Injury incidence rate ratio between OTC and OLV

The low incidence rate in the OTC might be attributed to athletes’ performance-optimization strategies such as tapering [25] and reduced training intensity to mitigate travel fatigue and jet lag [26]. Among a total of 97 OTC athletes, 40 (41%) stayed less than 5 days before moving to the OLV. Most athletes arrived late at night, proceeding directly to accommodations after flight. Upon leaving the OTC, many athletes transitioned to the OLV without additional training sessions.

### Burden

A study on Norwegian Olympic athletes from London 2012 to Tokyo 2020 showed a combined injury and illness incidence rate of 16.7 per 1,000 athlete-days [13]. Results of the Norwegian study revealed that the injury burden was 17.3 days per athlete-year, which was slightly greater than that of Team Korea at Paris 2024 (10.2 days per athlete-year). One study has investigated four-year medical records of Summer Olympic athletes in the Team Great Britain and reported a greater injury burden (3.6 injuries per 1000 athlete-days; injury burden 54.1 days per athlete-year and 1.3 illnesses per 1000 athlete-days; illness burden 10.4 days per athlete-year) [27]. However, since our study investigated a period when athletes were in their peak physical conditions, the burden was likely to be low. Interpreting illness burden requires caution, as zero recorded “time-loss” likely reflects athletes competing despite symptoms rather than no impact on performance in the high-stakes Olympic environment. Since training disruption predicts competitive failure [28], strong preventive measures against respiratory infections are vital for athlete health and performance at the LA 2028 Olympics.

## Limitations

This study comprehensively reported the incidence and detailed characteristics of injuries and illnesses among athletes from a single national delegation. Although this study offers robust methodological approaches, some limitations warrant careful acknowledgment. First, a small number of 96 cases of injuries and illnesses were reported though the result of the Game was outstanding for the small number of the participating athletes. This study included athletes from a moderate-size single delegation in Asia, which might limit its representation across all NOCs participating in the Olympic Games. Second, to determine a more precise incidence, prevalence surveys at the start of the event are needed [29]. It would be advantageous to extend information sources to athletic trainers to capture minor complaints, as observed in the Rio 2016 study conducted in Team Korea [20]. Finally, future research might need to include psychiatric conditions or female athlete health, which invariably demand a nuanced and careful approach [30, 31].

## Conclusions

Health problems in Team Korea athletes who competed for Paris 2024 were analyzed. Team Korea athletes exhibited higher incidences of injury and illness in Paris 2024 than in previous Olympic Games. Based on our findings, stakeholders can develop and implement targeted strategies to prevent injuries and illnesses. Injury and illness surveillance programs should continue to protect athletes’ well-being. By sharing our experience, we aim to encourage global prioritization of athlete health monitoring.

## Supplementary Information

Supplementary Material 1. List of Participating Sports Disciplines for Team Korea at the Paris 2024

## Data Availability

No datasets were generated or analysed during the current study.
