# Supplementary material for: Team Korea injury and illness surveillance at the 2024 Paris Olympic Games
Source: Inj Epidemiol. 2025 Sep 2;12:55. doi: 10.1186/s40621-025-00610-z (PMC12406425; doi:10.1186/s40621-025-00610-z)
Supplement: Supplementary file 1 — Supplementary Material 1. List of Participating Sports Disciplines for Team Korea at the Paris 2024 [file 40621_2025_610_MOESM1_ESM.pdf]

List of Participating Sports Disciplines for Team Korea at the Paris 2024

All Participating Sports (21 disciplines, 144 athletes)	Pre-Olympic Training Camp (13 disciplines, 97 athletes)	†Olympic Village (18 disciplines, 122 athletes)
Archery	Aquatics(Artistic Swimming, Swimming)	Archery
Aquatics(Artistic Swimming, Diving, Swimming)	Artistic Gymnastics	Aquatics(Artistic Swimming, Diving, Swimming)
Artistic Gymnastics	Athletics	Artistic Gymnastics
Athletics	Badminton	Athletics
Badminton	Boxing	Badminton
Boxing	Cycling	Boxing
Breaking	Fencing	Breaking
Climbing	Handball	Climbing
Cycling	Judo	Cycling
Equestrian	Modern Pentathlon	Equestrian
Fencing	Taekwondo	Fencing
Golf	Weightlifting	Handball
Handball	Wrestling	Judo
Judo		Modern Pentathlon
Modern Pentathlon		Table Tennis
Sailing		Taekwondo
Shooting		Weightlifting
Table Tennis		Wrestling
Taekwondo		
Weightlifting		
Wrestling		

†Note: Golf, Sailing, and Shooting, comprising 22 athletes, were excluded as they did not reside in the main Paris Olympic Village and were thus outside our medical support coverage.
